# Coordinately Regulated Alternative Splicing of Genes Involved in Cholesterol Biosynthesis and Uptake

**DOI:** 10.1371/journal.pone.0019420

**Published:** 2011-04-29

**Authors:** Marisa Wong Medina, Feng Gao, Devesh Naidoo, Lawrence L. Rudel, Ryan E. Temel, Allison L. McDaniel, Stephanie M. Marshall, Ronald M. Krauss

**Affiliations:** 1 Department of Atherosclerosis Research, Children's Hospital Oakland Research Institute, Oakland, California, United States of America; 2 Department of Pathology – Lipid Sciences, Wake Forest University Health Sciences, Winston-Salem, North Carolina, United States of America; University of Georgia, United States of America

## Abstract

Genes involved in cholesterol biosynthesis and uptake are transcriptionally regulated in response to cellular sterol content in a coordinated manner. A number of these genes, including 3-hydroxy-3-methylglutaryl coenzyme A reductase (*HMGCR*) and LDL receptor (*LDLR*), undergo alternative splicing, resulting in reductions of enzyme or protein activity. Here we demonstrate that cellular sterol depletion suppresses, and sterol loading induces, alternative splicing of multiple genes involved in the maintenance of cholesterol homeostasis including *HMGCR* and *LDLR*, the key regulators of cellular cholesterol biosynthesis and uptake, respectively. These changes were observed in both *in vitro* studies of the HepG2 human hepatoma derived cell line, as well as *in vivo* studies of St. Kitts vervets, also known as African green monkeys, a commonly used primate model for investigating cholesterol metabolism. These effects are mediated in part by sterol regulation of polypyrimidine tract binding protein 1 (PTBP1), since knock-down of PTBP1 eliminates sterol induced changes in alternative splicing of several of these genes. Single nucleotide polymorphisms (SNPs) that influence *HMGCR* and *LDLR* alternative splicing (rs3846662 and rs688, respectively), have been associated with variation in plasma LDL-cholesterol levels. Sterol-induced changes in alternative splicing are blunted in carriers of the minor alleles for each of these SNPs, indicating an interaction between genetic and non-genetic regulation of this process. Our results implicate alternative splicing as a novel mechanism of enhancing the robust transcriptional response to conditions of cellular cholesterol depletion or accumulation. Thus coordinated regulation of alternative splicing may contribute to cellular cholesterol homeostasis as well as plasma LDL levels.

## Introduction

Alternative splicing, the post-transcriptional editing process that can generate multiple mRNAs from a single gene, was once considered to be primarily a means of generating protein diversity [1]. However, more recently its role as a mechanism of regulating gene expression has been appreciated [2]. Functionally relevant alternative splicing has been reported in several genes involved in cellular cholesterol production and uptake [3], [4], [5], [6], [7], [8]. The gene encoding 3-hydroxy-3-methylglutaryl coenzyme A reductase (HMGCR), the enzyme that catalyzes the rate limiting step of cholesterol biosynthesis, undergoes alternative splicing of exon 13. Exon 13 skipping impairs enzymatic activity [3], [4], and also results in reduction of its sensitivity to inhibition by statins, a class of cholesterol lowering drugs that act as competitive inhibitors of HMGCR [4]. The LDL receptor (LDLR), which is primarily responsible for cellular uptake of LDL from plasma, has several splice variants, the two most common of which involve exon skipping: *LDLR4(-)* and *LDLR12(-)*, respectively. Both have been shown to be associated with reduced LDLR cell surface protein and LDL internalization, as well as hypercholesterolemia [6], [8]. Proprotein convertase subtilisin/kexin type 9 (PCSK9), which binds to the LDLR protein and induces its degradation, has a minor splice variant lacking exon 8 (*PCSK9 8(-))* that has no effect on LDLR protein turnover [5]. HMG-CoA synthase (HMGCS1), which catalyzes the reaction immediately before HMGCR, has a highly complex 5′ UTR that regulates translational efficiency and undergoes exon 2 skipping [7]. Mevalonate kinase, encoded by *MVK*, catalyzes the step immediately following HMGCR, and undergoes alternative splicing of exon 4 and/or 5, both of which disrupt the open reading frame [9]. Both *HMGCR* exon 13 skipping and *LDLR* exon 12 skipping are influenced by *cis*-acting SNPs [3], [4], [6] that have been found in genome-wide association analysis (GWAS) to contribute to inter-individual variation in plasma LDL-cholesterol in multiple independent populations [3], [6], [10], [11]. These findings suggest that alternative splicing of genes involved in cholesterol biosynthesis and receptor-mediated uptake has physiologically relevant effects on plasma LDL-cholesterol.

Polypyrimidine tract binding protein (PTBP1) antagonizes the function of the essential splicing factor U2AF in the recognition of the 3′ splice site [12], and thus acts as a negative splicing regulator of numerous pre-mRNAs [13], [14], [15]. PTBP1 has been postulated to modulate *LDLR* mRNA stability by binding to the 3′ UTR [16], and therefore it is possible that such an interaction may also influence alternative splicing.

Since *HMGCR, LDLR, HMGCS1, MVK* and *PCSK9* are among the genes whose transcription is regulated in a coordinated fashion by SREBP in response to sterols [17], [18], we sought to determine if the relative levels of the alternatively spliced versus full-length transcripts are also subject to sterol regulation. Here we provide evidence that the relative amounts of alternatively spliced transcripts of *HMGCR, LDLR, HMGCS1, MVK* and *PCSK9* in hepatocytes (HepG2), human immortalized lymphoblast cell lines, and livers from cholesterol-fed African Green monkeys, are reduced under conditions of sterol depletion, and induced under conditions of sterol loading. These results implicate alternative splicing in the coordinated regulation of expression of genes involved in cholesterol biosynthesis and uptake.

## Results

### The relative expression level of alternatively spliced to full-length HMGCR is sterol regulated

**Figure 1:**
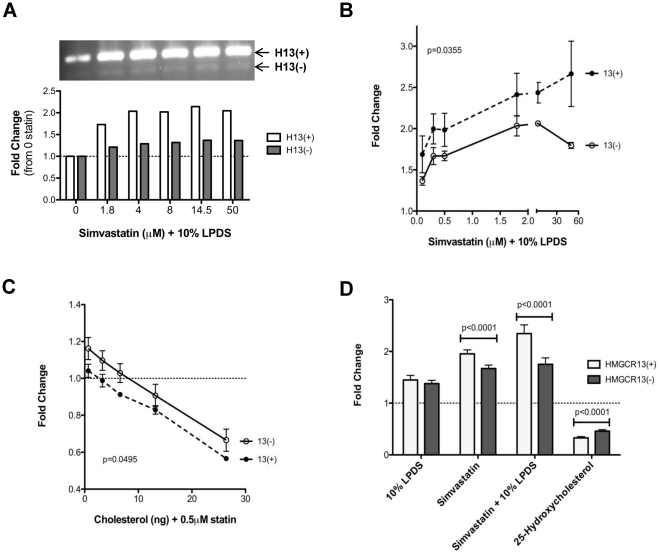
HMGCR alternative splicing is sterol regulated. **A**) Fold change in *HMGCR13(+)* and *13(−)* transcripts with conditions of sterol depletion. IMR-90 cells were incubated with varying concentrations of activated simvastatin in the absence of exogenous cholesterol for 24 hours. RT-PCR with primers spanning exons 12 to 14 was used to amplify both the *HMGCR13(+)* and *13(−)* transcripts, and band density was quantified. One representative sample is shown. B) Fold change in *HMGCR* transcript with conditions of sterol depletion. HepG2 cells were incubated with varying concentrations of activated simvastatin in the absence of exogenous cholesterol for 24 hours, and changes in transcript levels were quantified by real time PCR using splice variant specific assays, n = 8. **C**) Fold change in *HMGCR* transcript with condition of sterol loading. HepG2 cells were incubated with 0.5 µM simvastatin with varying concentrations of LDL-cholesterol for 24 hours and changes in transcript levels were quantified by real time PCR, n = 8. **D**) Fold change in *HMGCR* transcript with varying conditions of sterol depletion and loading. Immortalized lymphoblast cell lines were incubated with either 10% FBS (control), 10% LPDS, 0.5 µM simvastatin + 10% FBS, 0.5 µM simvastatin + 10% LPDS or 1 µg/ml 25-hydroxycholesterol + 10% LPDS for 24 hours, n = 26. *HMGCR* transcripts were quantified by real time PCR and fold change was calculated from the 10% FBS incubated samples. Repeated measures MANOVA was used to identify statistical significance in the difference in fold changes between the *13(+)* and *13(−)* transcripts across the different incubation conditions (A) and (B), and paired t-tests were used to identify statistically significant differences in fold changes of *HMGCR13(+)* versus *13(−)* per incubation condition (C). Values plotted are mean ± s.e.m.

To determine if the ratio of *HMGCR* alternatively spliced to full-length transcripts is sterol regulated, both the full-length transcript, *HMGCR13(+)*, and the alternatively spliced form, *HMGCR13(−)*, were measured in HepG2 and IMR-90 cells after sterol depletion by 24 hour incubation with varying concentrations of simvastatin (0.1 to 50 µM), and 10% lipoprotein deficient serum (LPDS). As shown in Figure 1A, extreme cholesterol depletion (statin + LPDS) of IMR-90 cells up-regulated both *HMGCR13(+)* and *13(−)* transcripts, however, the relative induction of *HMGCR13(−)* was substantially lower than the *13(+)* transcript. Since quantitative real time PCR has a much larger dynamic range for quantitation of mRNA transcripts compared to measurements of band density, all subsequent transcript quantification was performed by splice variant-specific real time PCR as described in the Methods. Cholesterol depletion of HepG2 cells induced *HMGCR13(−)* 16–31% (20% average) less than the *13(+)* transcript, p = 0.036 **(** **Figure 1B**). In addition, provision of LDL-derived cholesterol to HepG2 cells incubated with 0.5 µM simvastatin (**Figure 1C**) resulted in 10–17% (12% average) greater suppression of the *HMGCR13(+)* transcript in comparison to the *HMGCR13(−)* transcript (p = 0.049), suggesting that sterol loading induced *HMGCR* alternative splicing. These effects were confirmed in 26 immortalized lymphoblast cell lines incubated with either 10% FBS, 10% LPDS, 0.5 µM simvastatin, 0.5 µM simvastatin + 10% LPDS, or 1 µg/ml 25-hydroxycholesterol + 10% LPDS. As shown in **Figure 1D**, *HMGCR13(−)* was induced 15% less than the *HMGCR13(+)* transcript in the simvastatin incubated cells lines, and 25% less in the simvastatin + LPDS incubated cells, while sterol loading with 25-hydroxycholesterol suppressed *HMGCR13(−)* 34% less than the *13(+)* transcript (p<0.0001 for each treatment). Suppression of *HMGCR* alternative splicing with extreme sterol depletion (statin + LPDS) was also confirmed in freshly isolated peripheral blood mononuclear cells (n = 7, data not shown) demonstrating that this phenomenon is not unique to transformed cells.

### Sterols regulate the relative ratio of alternatively spliced to full-length transcripts in multiple genes involved in cholesterol biosynthesis and uptake

**Figure 2:**
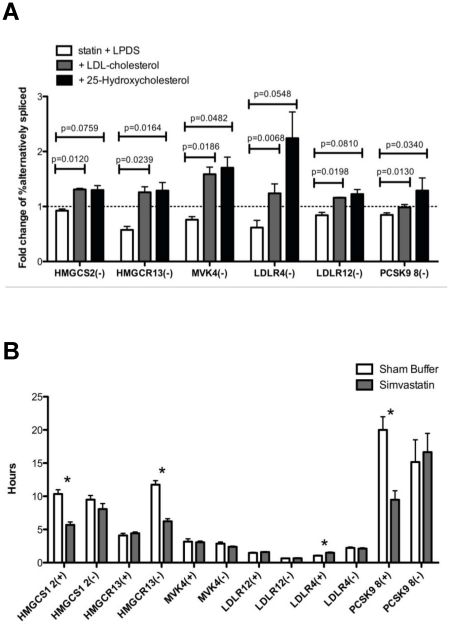
Multiple genes involved in regulating cholesterol homeostasis are subject to sterol regulated alternative splicing. **A**) HepG2 cells were incubated with either 2.0 µM simvastatin +10% LPDS or sham buffer with 10% FBS. After 24 hours, either 50 µg/ml LDL-cholesterol or 1 µg/ml 25-HC were added to the statin treated sample and incubated for an additional 24 hours, n = 6. Fold changes were calculated from the cells incubated with 10% FBS only. **B**) Splice variant specific changes in transcript half-life in response to sterol depletion. HepG2 cells were incubated with either 2.0 µM simvastatin + 10% LPDS or sham buffer with 10% FBS in replicate 6-well plates. After 24 hours, 1 µg/ml Actinomycin D was added, and individual wells were collected after 0, 0.5, 1, 2, 4, 6, 24 and 48 hours, n = 12. mRNA quantity values were log transformed and plotted versus time. Linear regression was used to calculate the slope of the resulting line, and mRNA half-life was calculated as: *t*_1/2_ (h)  =  ln2/(−2.303× slope). P-values were calculated as paired two tailed t-tests. Values plotted are mean ± s.e.m.

Given the strong evidence for sterol regulation of *HMGCR* splice variants, we tested whether there are also sterol-regulated changes in the relative amounts of alternatively spliced to full-length transcripts of other key regulatory genes involved in cholesterol metabolism - *LDLR, HMGCS1, MVK*, and *PCSK9.* HepG2 cells were incubated with 2.0 µM simvastatin + 10% LPDS or sham buffer + 10% FBS for 24 hours, after which either 50 µg/ml LDL-cholesterol or 1 µg/ml 25-HC were added and incubated for an additional 24 hours. These genes are known to be transcriptionally responsive to sterols [17], and as expected, expression of each was up-regulated by statin exposure, effects that were reversed by the addition of LDL-cholesterol or 25-HC (data not shown). Similar to *HMGCR13(−),* all splice variants of these genes -*LDLR4(−), LDLR12(−), MVK4(−), HMGCS1 2(−)*, and *PCSK9 8(−)* - showed evidence of sterol regulation (**Figure 2A**). Sterol depletion suppressed the relative amounts of alternatively spliced to full-length transcripts by 8–50%, while addition of either LDL-cholesterol or 25-hydroxycholesterol increased these ratios by 16–124% in comparison to standard culture conditions (10% FBS). These phenomena were confirmed in immortalized lymphoblast cell lines (n = 6, data not shown).

### Sterol regulation of mRNA stability does not differ between full-length and alternative spliced transcripts

To determine if sterol-induced changes in the ratios of alternative spliced to full-length transcripts were due to transcript-specific differences in mRNA decay rates, HepG2 cells were pre-incubated with either 2.0 µM simvastatin + 10% LPDS or sham buffer + 10% FBS for 24 hours, after which 1 µg/ml actinomycin D was added to the media (n = 12 experiments). Although statin treatment did not affect stability of the *HMGCR13(−)* transcript, the half-life of the *HMGCR 13(−)* transcript was 53% lower than in the sham treated sample (6.2±0.4 hr versus 11.8±0.6 hr respectively, p<0.00001, **Figure 2B**). Sterol depletion also increased the half-life of the *LDLR4(+)* transcript (1.1±0.1 hr sham versus 1.5±0.1 hr statin, p = 0.0007) but did not affect stability of the *LDLR4(−)* transcript. These results indicate that differential changes in message stability of *LDLR4(−)* and *HMGCR13(−)* in comparison to their full length counterparts following statin treatment may contribute to a relative reduction in their cellular levels. In contrast, there were no changes in transcript half-life of *MVK4(+), MVK4(−), LDLR12(+)* or *LDLR12(−).* Paradoxically, both the full length *HMGCS1* and *PCSK9* transcripts had significantly reduced transcript half-lives after sterol depletion while there was no change in mRNA stability of their respective alternatively spliced variants. These results strongly suggest that the sterol-induced changes in the ratio of alternative spliced to full-length *HMGCS, MVK, LDLR12,* and *PCSK9* are not due to splice variant specific differences in transcript stability.

### LDLR alternative splicing is induced in cholesterol fed African Green monkeys

**Figure 3:**
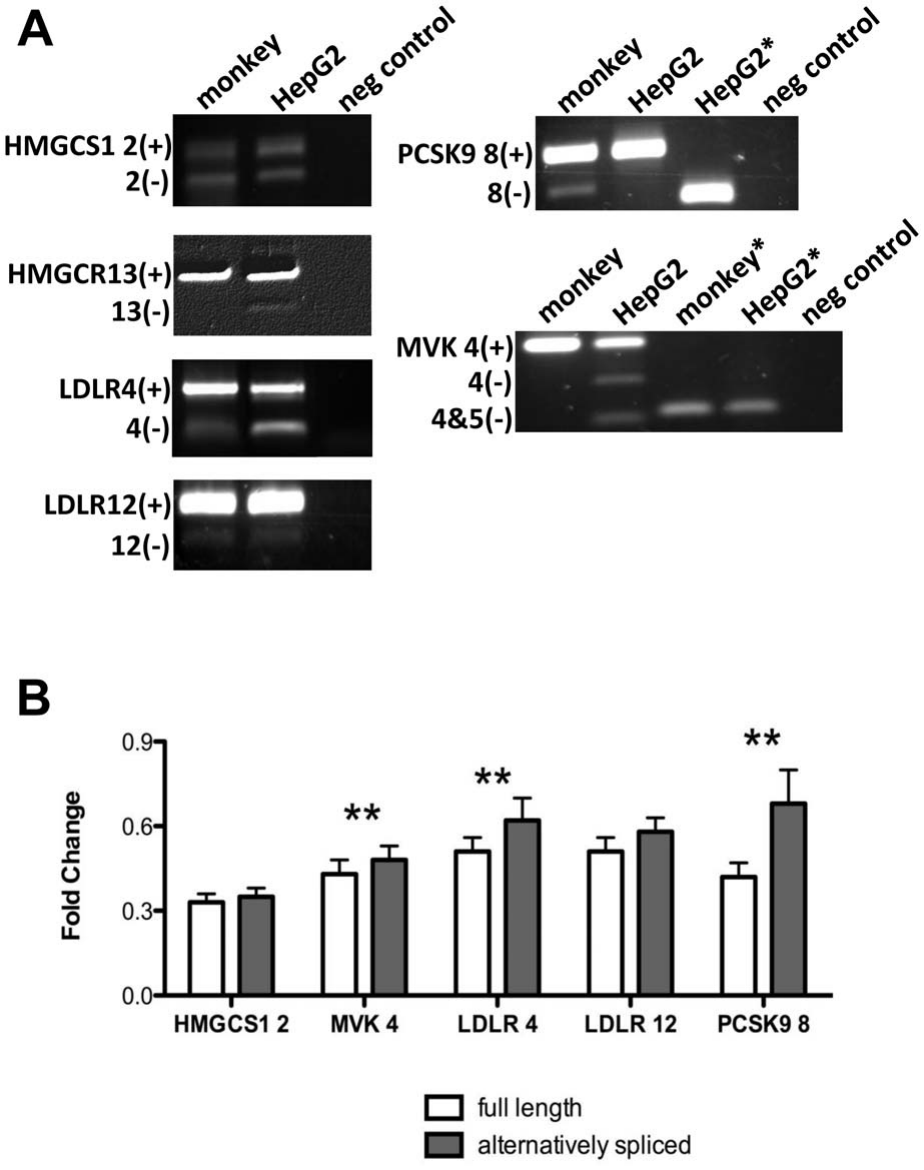
Validation of sterol-induced changes in alternative splicing in cholesterol fed African Green monkeys. **A**) RT-PCR was performed with primers spanning alternatively spliced exons in *HMGCS1, HMGCR, MVK, LDLR* and *PCSK9* with cDNA derived the liver of an African Green monkey or HepG2 cell line (human control). *The *PSCK9 8(−)* and *MVK4(−)* transcripts are minor species in humans and monkeys respectively and difficult to visualize via RT-PCR, thus PCR was performed using primers that specifically amplify the alternatively spliced transcripts. **B**) Full length and alternative spliced *HMGCS1, MVK, LDLR* and *PCSK9* transcripts were quantified in liver biopsies obtained from monkeys with (n = 28) and without (n = 23) cholesterol supplementation. Fold changes were calculated for each cholesterol fed animal as the transcript quantity value divided by the average of all control diet fed animals. Paired t-tests were used to identify statistically significant differences in the magnitude of fold change of the full-length versus alternatively spliced transcripts. **Indicates p<0.05. Values plotted are mean ± s.e.m.

To determine if the splice variants of interest were expressed in liver from the African Green monkey, we used RT-PCR with primers spanning the alternatively spliced exons of interest, and DNA sequencing, to identify and confirm expression of *HMGCS1 2(−), MVK4(−), LDLR4(−), LDLR12(−),* and *PCSK9 8(−),* (**Figure 3a**). *HMGCR13(−)* was not detected. The splice variants were quantified along with the *HMGCS1 2(+), MVK4(+), LDLR4(+), LDLR12(+)* and *PCSK9 8(+)* transcripts in liver biopsies of monkeys fed either a cholesterol-supplemented or control diet. As expected, all transcripts were down-regulated by cholesterol feeding, but *MVK4(−), LDLR4(−)* and *PCSK9 8(−)* transcripts were suppressed less than their full-length counterparts (p<0.05, n = 28, **Figure 3b**). Although *HMGCS1 2(−)* and *LDLR12(−)* also appeared to be suppressed less than *HMGCS1 2(+)* and *LDLR12(+)*, these differences did not achieve statistical significance. Additional analyses suggested a correlation of change in hepatic total cholesterol and cholesterol ester content with the magnitude of change in alternative splicing, in that animals with greater increases in hepatic total cholesterol and cholesterol ester also had greater increases in *% LDLR12(−)* (**Figure S1**).

### Alternative splicing is not regulated by non-sterol end-products of the mevalonate pathway

**Figure 4:**
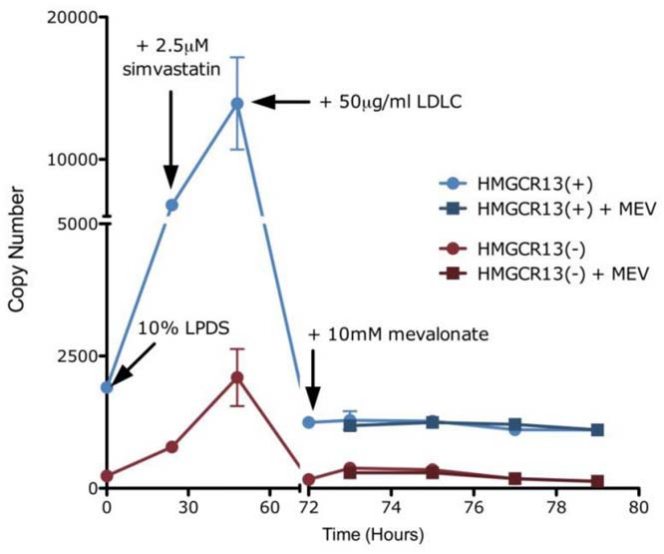
Statin induced changes in *HMGCR* alternative splicing are sterol mediated. IMR-90 cells were incubated with 10% LPDS for 24 hours in duplicate, after which 2.5 µM simvastatin was added to the media. After 24 hours, 50 µg/ml LDL-cholesterol was added and incubated for an additional 24 hours. 10 mM mevalonate was added to one set of cells, while 50 µg/ml LDL-cholesterol was added to the second set of cells. Aliquot were removed after 1, 2, 4 and 6 hours, and *HMGCR13(+)* and *13(−)* was quantified by qPCR. The entire experiment was performed in triplicate. Values plotted are mean ± s.e.m.

Since statin treatment blocks production of both cholesterol and other non-sterol end-products of the mevalonate pathway such as isoprenoids, we sought to determine if changes in alternative splicing are mediated primarily by one or more of these end-products. Because non-sterol regulation of HMGCR has traditionally been studied in fibroblasts [19], we incubated early passage normal human diploid fibroblasts (IMR-90) for 24 hours with 2.0 µM simvastatin + 10% LPDS in duplicate, after which LDL-C (50 µg/ml) was added to the media. After 24 hours, 10 mM mevalonate was added to one set of cell lines, while the other received an additional dose of cholesterol, and aliquots were removed over the course of 6 hours. As expected, *HMGCR13(+)* transcript levels did not differ between the cell lines treated with LDL-C alone versus those treated with LDL-C plus mevalonate (**Figure 4**), consistent with previous reports that *HMGCR* transcriptional regulation is mediated by sterols and not by non-sterol products of the mevalonate pathway [20]. *HMGCR13(−)* transcript levels were also unchanged by the addition of mevalonate, indicating that statin suppression of *HMGCR* alternative splicing is regulated by changes in sterol production. Furthermore, there was no evidence for non-sterol regulation of amounts of either *LDLR4(−)* or *12(−)* (data not shown), suggesting that sterols are responsible for coordinated changes in alternative splicing of genes involved in cholesterol metabolism.

### Sterol regulation of splicing occurs prior to transcriptional response

**Figure 5:**
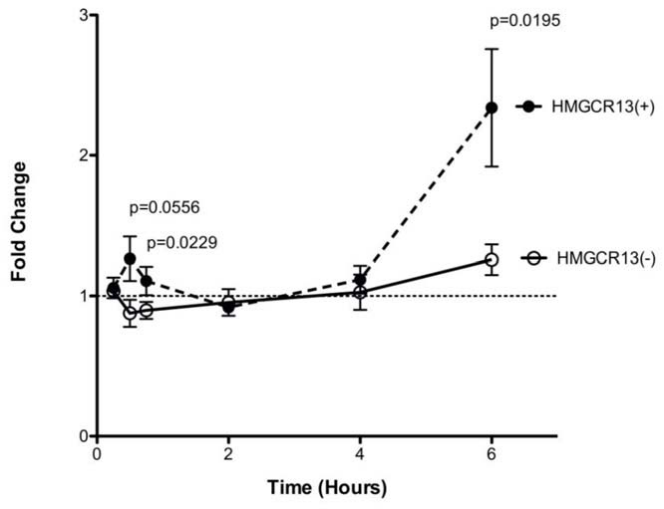
Transcriptional versus splicing regulation in HepG2 cells. Time course analysis of *HMGCR* gene expression in HepG2 cells incubated with 2.0 uM simvastatin +10% LPDS, or placebo + 10% FBS, n = 5. Change in *HMGCR* alternative splicing occurs within 30 min minutes post-treatment as indicated by an immediate down-regulation of *HMGCR13(−)*, open circles, and corresponding up-regulation of *HMGCR13(+)*, solid circles. Data were calculated as the ratios of the fold changes of the statin versus placebo treated sample at each individual time point in comparison to 0 minutes. Values plotted are mean ± s.e.m.

Although pre-mRNA spicing and transcription are related processes, we sought to determine if there was evidence for regulation of alternative splicing prior to a detectable change in overall transcription. *HMGCR13(+)* and *13(−)* transcripts were quantified in HepG2 incubated with either 2.0 µM simvastatin + 10% LPDS or sham buffer + 10% FBS over the course of 6 hours (n = 5) (**Figure 5**). Decreased %*HMGCR13(−)* occurred within 30–45 minutes as a result of up-regulation (1.3±0.16 fold) of the *HMGCR13(+)* transcript with a corresponding down-regulation (0.9±0.05 fold) of the *HMGCR13(−)* transcript. This effect subsided within 2 hours post-exposure and was followed by a second phase of transcriptional up-regulation of both *HMGCR13(+)* and *13(−)*. Similar results were obtained with immortalized lymphoblast cell lines (n = 6, data not shown). This early change in alternative splicing was not seen for *LDLR* exon 4 or 12 skipping, or for *MVK* exon 4 skipping (data not shown).

### Sterol regulated alternative splicing varies in a coordinated fashion among individuals

In 24 immortalized lymphoblast cell lines incubated with either 2.0 µM simvastatin or sham buffer there was a positive correlation between magnitude of change in %*HMGCR13(−)* and %*LDLR12(−)* (p = 0.02, r^2^ = 0.21) and a weaker although non-significant (p = 0.19) relationship between *%HMGCR13(−)* and *%LDLR4(−)*. There was no evidence for a correlation between *%HMGCR13(−)* fold change and either *LDLR12(+)* or *4(+)* fold change (data not shown).

**Figure 6:**
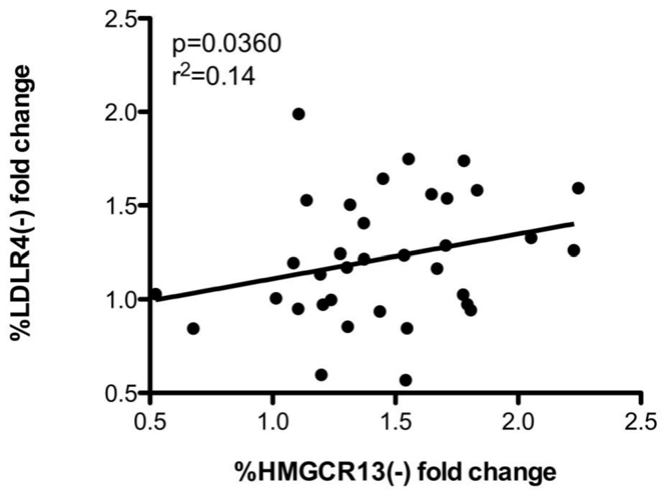
Sterol induced changes in *HMGCR* and *LDLR* alternative splicing are correlated. Immortalized lymphoblast cell lines were incubated for 24 hours with either 1 µg/ml 25-hydroxycholesterol + 10% LPDS or standard culture conditions (10% FBS), n = 31. Fold changes were calculated as percent alternative spliced in the 25-HC treated sample divided by the 10% FBS treated sample, and statistical significance was calculated using linear regression.

A similar correlation in the degree of induction of *HMGCR* and *LDLR* alternative splicing was seen after sterol loading in 31 lymphoblast cell lines incubated with 1 µg/ml 25-hydroxycholesterol. As expected, *%HMGCR13(−), %LDLR12(−)* and *%LDLR4(−)* were up-regulated by sterol loading (1.5±0.1 fold, 1.5±0.1 and 1.2±0.1 fold respectively). The changes in %*HMGCR13(−)* and *%LDLR4(−)* were correlated (p = 0.036, **Figure 6**) with a weaker and non-significant (p = 0.17) relationship between *%HMGCR13(−)* and *%LDLR12(−).* Again, there was no relationship between *%HMGCR13(−)* fold change and either *LDLR4(+)* or *12(+)* fold change.

### PTBP1 mediates sterol regulated changes in alternatively spliced transcripts

**Figure 7:**
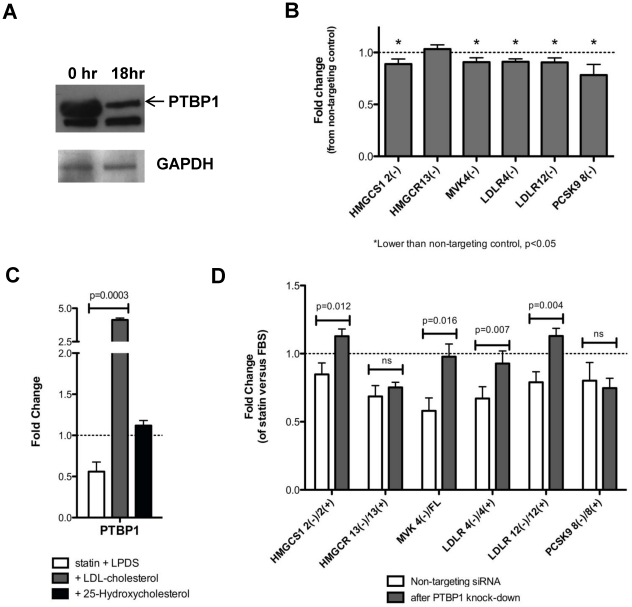
PTBP1 mediates sterol regulation of alternative splicing of genes involved in cholesterol biosynthesis and uptake. **A**) Representative western blot of HepG2 cells before and after 18 hr transfection with *PTBP1* Silence Select siRNA. **B**) Effect of PTBP1 knock-down on the relative ratios of full-length to alternatively spliced transcripts. HepG2 cells were transfected with either *PTBP1* Silence Select siRNA or a non-targeting siRNA control for 18 hours, n = 12 Fold changes were calculated as percent alternatively spliced in the cells transfected with the PTBP1 specific siRNA divided by the percent alternatively spliced in cells transfected with the non-targeting negative control. **C**) HepG2 cells were treated as described in Figure 3. Fold change in *PTBP1* gene expression was calculated from cells incubated with sham buffer + 10% LPDS. Statistically significant differences were calculated using two-tailed paired t-tests, n = 6. **D**) HepG2 cells were transfected with either a siRNA targeted to PTBP1 or a non-targeting negative control in duplicate. After 18 hours, incubation media was refreshed to include either 2.0 µM simvastatin + 10% LPDS or sham buffer + 10% FBS and cells were incubated for an additional 24 hours, n = 6. Statin induced fold changes in percent alternatively spliced were calculated independently in the PTBP1 siRNA versus non-targeting negative control samples as the value measured in the statin incubated sample divided by the value measured in the sham incubated sample. All values shown are mean ± s.e.m. FL  =  full length.

PTBP1 is a splicing regulator that promotes alternative splicing [13], [14], [15] and has been previously shown to bind the *LDLR* transcript [16]. Since changes in alternative splicing appear to be coordinately regulated, we tested if PTBP1 influences alternative splicing of *LDLR* as well as other genes involved in cholesterol biosynthesis and uptake, HepG2 cells were transfected with a Silence Select siRNA specific for *PTBP1* or a non-targeting negative control, and splice variants were quantified by qPCR (n = 24). Transfection with the *PTBP1* specific siRNA reduced *PTBP1* gene expression by 68.0±0.03%, and protein expression by 66% (**Figure 7A**). PTBP1 knock-down resulted in reduction of the relative levels of *LDLR4(−), LDLR12(−), HMGCS1 2(−), MVK4(−),* and *PCSK9 8(−)* by 9–23%, (p<0.05, **Figure 7B**). The non-targeting siRNA did not affect *PTBP1* gene expression or alternative splicing of any of the genes tested (data not shown). Interestingly, *HMGCR13(−)* was unchanged by *PTBP1* knock-down, indicating that this splicing event is regulated by other mechanisms.

Since PTBP1 binding to the *LDLR* 3′ UTR has been shown to regulate the half-life of several transcripts [16], we sought to determine if the changes in the relative abundance of alternatively spliced transcripts after PTBP1 knock-down were due to transcript specific differences in mRNA half-life. HepG2 cells were transfected with either the non-targeting control or PTBP1-specific siRNA for 18 hrs, and subsequently treated with actinomycin D (n = 12). There were no splice variant-specific differences in transcript stability with PTBP1 knock-down (**Figure S2**), indicating that the changes in the relative ratios of alternatively spliced to full-length transcripts were most likely due to direct effects on alternative splicing of these genes.

Sterol regulation of *PTBP1* was assessed in HepG2 cells exposed to sterol depletion for 24 hours after which either LDL-cholesterol or 25-hydroxycholesterol was added (conditions described in Figure 2). *PTBP1* mRNA expression was reduced to 0.6±0.2 fold of control by sterol depletion (statin + LPDS), while incubation with sterols reversed this effect by up-regulating *PTBP1* 4.1±0.1 fold (**Figure 7C**), demonstrating that *PTBP1* is transcriptionally responsive to sterols.

To determine if PTBP1 mediates sterol-regulated changes in alternative splicing, HepG2 cells were incubated with either a siRNA specific for PTBP1 or a non-targeting negative control for 18 hours, after which either 2.0 µM simvastatin or placebo was added for an additional 6 hours (n = 9). PTBP1 knock-down attenuated the suppression of alternative splicing with sterol depletion (**Figure 7D**). The ratios of alternatively spliced to full-length transcripts of *HMGCS1, LDLR* and *MVK* were reduced in the cells transfected with the non-targeting siRNA after sterol depletion, but not after PTBP1 knock-down. Notably, sterol induced changes in *HMGCR13(−)/13(+)* or *PCSK9 8(−)/8(+)* were not affected by PTBP1 knock-down. PTBP1 knock-down also attenuated statin-induced transcriptional up-regulation of *HMGCS, MVK* and *LDLR12(+)*, but had no effect on *LDLR4(+)* (**Figure S3**). Taken together, these results demonstrate that sterol induced changes in the relative levels of alternatively spliced to full length transcripts in multiple genes is mediated in part by down-regulation of PTBP1.

### Sterol depletion and loading blunts genetic regulation of HMGCR and LDLR alternative splicing

**Figure 8:**
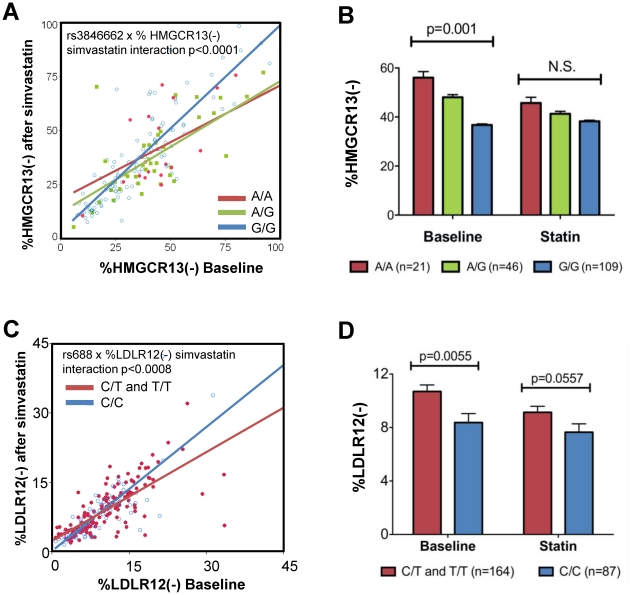
Interaction between genetic and non-genetic regulators of *HMGCR* and *LDLR* alternative splicing. A) Correlation of %*HMGCR13(−)* expressed after 24 hour treatment with 2.0 µM simvastatin versus sham buffer (baseline value) in 134 immortalized lymphoblast cell lines split by rs3846662 genotype. The p-value indicates that the interaction between %*HMGCR13(−)* and rs3846662 genotype was statistically significant. B and D) %*HMGCR13(−)* and %*LDLR12(−)* at baseline conditions and after statin treatment split by rs3846662 or rs688 genotype respectively. P-values were calculated using a two-tailed paired t-test. Values shown are mean ± s.e.m. C) Correlation of %*LDLR12(−)* expressed after treatment with simvastatin versus after treatment with placebo (baseline value) in 251 immortalized lymphoblast cell lines. A dominant genetic model was applied since C/T and T/T cell lines behaved identically.

Although statin treatment suppresses both *HMGCR13(−)* and *LDLR12(−)* alternative splicing, the absolute amount of these transcripts expressed after statin incubation vs. baseline remains tightly and positively correlated among immortalized lymphoblast cell lines (p<0.0001, r^2^ = 0.70, n = 173 and p<0.0001, r^2^ = 0.63, n = 251 respectively). This suggests intrinsic differences in factors regulating alternative splicing of these genes among the cell lines. Since both *HMGCR13(−)* and *LDLR12(−)* are genetically regulated by *cis-*acting SNPs (rs3846662 and rs688 respectively [3], [4], [6]), we tested for interactions between genetic and non-genetic regulation of alternative splicing. Interestingly there appears to be an interaction between *HMGCR* rs3846662, the intron 13 SNP known to directly influence exon 13 alternative splicing [3], [4], and the degree of statin suppression of exon 13 skipping, since this phenomenon was only seen in cell lines that carry at least one copy of the “A” allele of rs3846662 (**Figure 8A**). Consequently, the relationship between rs3846662 and %*HMGCR13(−)* expression was blunted by statin treatment (interaction p<0.0001, **Figure 8B**). Similar results were seen with rs688, an exon 12 SNP that regulates *LDLR* exon 12 alternative splicing, and %*LDLR12(−),* where sterol suppression of exon 12 skipping occurred only in cell lines with at least one copy of the “T” or minor allele (**Figure 8C**). Using a dominant genetic model, rs688 genotype remained significantly associated with %*LDLR12(−)* after statin treatment, however this relationship was attenuated compared to that with baseline %*LDLR12(−)* (**Figure 8D**). Given the sterol by genotype interaction in regulation of alternative splicing, we genotyped the HepG2 cell line and found that it was heterozygous for the minor allele of rs3846662 (A/C) and homozygous for the major allele of rs688 (C/C).

## Discussion

The genes involved in cellular cholesterol biosynthesis and uptake are known to be coordinately regulated by the SREBP/Insig/SCAP system in response to sterols such that these pathways are induced and suppressed as a whole [17]. Here we provide evidence that orchestrated regulation of *HMGCR* and *LDLR,* the two key regulatory genes responsible for cholesterol biosynthesis and uptake, respectively, also occurs at the level of alternative splicing. Furthermore, other genes in the pathway of cholesterol biosynthesis including *HMGCS1* and *MVK,* as well as another key gene involved in cholesterol uptake, *PCSK9,* are subject to similar regulation, suggestive of a pathway level effect. Although coordinated regulation of the cholesterol biosynthetic pathway has also been seen at the level of enzyme activity and/or protein amount [21], [22], [23], [24], these changes have been attributed largely to mechanisms that influence protein synthesis such as SREBP-induced gene expression. Coordinated regulation of pre-mRNA splicing of multiple genes by an external stimulus has been recently demonstrated in the case of insulin, which has been found to affect splicing of over 150 genes [25]. Moreover, orchestrated changes in alternative splicing have been shown in several biological processes including apoptosis [26].

Alternative splicing of *HMGCR*, *LDLR*, *MVK,* and *PCSK9* reduces protein or enzyme activity [3], [4], [5], [6], [8], [9], [27], [28], [29]. In addition, we found here that alternative splicing of the 5′ UTR of *HMGCS1* reduces the half-life of the transcript, consistent with the likelihood that this process also results in decreased HMGCS1 protein levels. We found that cellular cholesterol deficiency reduced the ratio of alternatively spliced to full-length transcripts (increasing enzyme or protein activity) while cholesterol accumulation increased this ratio (decreasing enzyme or protein activity). These data strongly suggest that modulating the ratio of full-length to alternatively spliced transcripts is a generalized mechanism for regulating expression of genes involved in cholesterol biosynthesis and uptake. Moreover, we found evidence for *in vivo* operation of this mechanism by showing that cholesterol feeding influences alternative splicing of hepatic *HMGCS1, MVK, LDLR* and *PCSK9* in African Green monkeys. Although *in vivo* sterol regulation of *HMGCR13(−)* could not be confirmed in this species due to lack of detectable hepatic expression, we have identified its presence in other non-human primates (*personal communication M.W.M)*. As described in the supplementary material, the association of the magnitude of alternative splicing with hepatic content of cholesterol ester but not free cholesterol likely represents the role of the former as the storage form of excess diet-derived cholesterol, while multiple homeostatic mechanisms operate to limit increases in cellular free cholesterol.

Co-regulation of alternative splicing of multiple genes in pathways affecting cholesterol metabolism may provide a means to quickly modulate or fine-tune the effects of SREBP-mediated transcriptional regulation of intracellular cholesterol content. Indeed, our results demonstrate that sterol-induced changes in *HMGCR* alternative splicing can occur prior to detectable transcriptional response. Although our incubations with IMR-90 fibroblasts suggest that *HMGCR* alternative splicing is not regulated by non-sterol end-products of the mevalonate pathway, these results do not exclude the possibility of more immediate regulation (within 45 minutes post-treatment) of *HMGCR* alternative splicing by products other than cholesterol generated from the mevalonate pathway.

Coordinated regulation of alternative splicing across multiple genes suggests that there are shared mechanisms responsible for generating this response. One possibility for such a mechanism is suggested by our finding that siRNA knockdown of PTBP1, an RNA binding protein and negative splicing regulator previously shown to bind the *LDLR* gene [16], reduces the relative levels of *LDLR, HMGCS1, MVK*, and *PCSK9* splice variants. Although these changes were relatively modest, it was recently reported that siRNA knock-down of *PTBP1* results in the up-regulation of *PTBP2.* Since PTBP1 and PTBP2 have similar functional effects, dramatic changes in splicing are seen only when both *PTBP1* and *PTBP2* are knocked down [30]. Thus, our results indicate that modulation of PTBP1 reduces the level of alternatively spliced mRNA despite the compensatory up-regulation of *PTBP2,* which also occurs when *PTBP1* is down-regulated in response to sterol depletion (*personal communication, M.W.M. and F.G*.). Moreover, the attenuation of *HMGCS1, MVK* and *LDLR12(+)* transcriptional response to statin after PTBP1 knock-down suggests that these genes are also subject to transcriptional regulation by PTBP1, consistent with previous reports that PTBP1 can bind and activate promoters [31], [32]. However, the specificity of this activity is different from its effects on mRNA stability, as demonstrated by the robust effects of PTBP1 knock-down on *LDLR* exon 4 skipping, but not on statin-induced expression of the *LDLR4(+)* transcript.

We also found that *PTBP1* gene expression is sterol regulated since sterol depletion reduced *PTBP1* expression and this was reversed by sterol loading. These results are consistent with recent reports demonstrating that PTBP1 protein is up-regulated *in vivo* in LDLR^−/−^ mice fed a Western versus chow diet [33]. Furthermore, *PTBP1* knock-down eliminated sterol induced regulation of alternative splicing of *LDLR, HMGCS1* and *MVK.* These findings, together with the down regulation of *PTBP1* gene expression by sterol depletion, are consistent with the likelihood that regulation of *PTBP1* mediates sterol-induced changes in the magnitude of alternative splicing of *LDLR, HMGCS1,* and *MVK.* Although PTBP1 knock-down reduced *PCSK9 8(−)/8(+),* it did not influence sterol induced change in this ratio; in addition, *PTBP1* does not appear to regulate *HMGCR*, suggesting the involvement of other splicing factors in generating these variants. This is consistent with previous reports that PTBP1 knock-down did not alter either *HMGCR* or *SREBP2* transcript levels [16]. Given the fact that PTBP1 has been shown to regulate alternative splicing of numerous other genes not previously implicated in cholesterol metabolism [13], [14], [15], our findings raise the possibility that sterols influence alternative splicing on a more global scale beyond the genes described here. However, in the absence of changes in transcriptional response, the physiological effects of changes in alternative splicing may be minimal.

Although many of the coordinated changes in the relative ratios of alternatively spliced to full-length transcripts can be attributed at least in part to PTBP1 mediated changes in alternative splicing, we found that splice variant specific regulation of mRNA half-life also occurs. The reduction in %*HMGCR13(−)* with sterol depletion is likely due to a combination of both transcript-specific changes in half-life and direct effects on exon 13 skipping. We showed that sterol depletion specifically reduced the half-life of the *HMGCR13(−)* transcript, but had no effect on the half-life of *HMGCR13(+)*. However, we also found that the absolute level of *HMGCR13(−)* drops while *HMGCR13(+)* increases within 30 minutes of statin exposure, an effect not likely attributable to changes in transcript stability. In addition, as described further below there was an interaction between statin-induced changes in %*HMGCR13(−)* and rs3846662, a SNP that directly regulates exon 13 skipping, demonstrating that there are also sterol-induced changes in the process of alternative splicing. Although splice variant-specific changes in mRNA stability and direct regulation of alternative splicing can each contribute to the overall changes in the relative abundance of alternatively spliced transcripts, regulation at the level of mRNA stability was only identified in two of the transcripts studied, *HMGCR13(−)* and *LDLR4(−),* while changes in alternative splicing were evident for all six transcripts studied, consistent with a pathway level effect.

Using a repository of immortalized lymphoblast cell lines, we demonstrated that the magnitude of sterol-induced changes in both suppression and induction of *HMGCR* and *LDLR* percent alternatively spliced transcripts varied widely among individual cell lines, but that these changes were strongly correlated among the cell lines. Although we previously reported that *HMGCR13(+)* and *13(−)* were induced to a similar degree in 172 immortalized lymphoblast cell lines incubated with either 1.8 or 14.5 µM activated simvastatin in the presence of 10% FBS (24 hours) [4], additional data (total n = 185) revealed that the *13(−)* transcript was induced slightly less (5.1%, p = 0.039) than the *13(+)* transcript. Since these cells were exposed to identical incubation conditions, variation in response suggests that there is a genetic component in the regulation of alternative splicing. *Cis-*acting SNPs in both *HMGCR* and *LDLR*, rs3846662 and rs688, have been shown to regulate exon 13 and exon 12 skipping respectively [3], [6]. Notably, there was evidence for a strong interaction between genetic and sterol regulation of alternative splicing since sterol depletion blunted the relationship between rs3846662 and *%HMGCR13(−)*, and rs688 with *%LDLR12(−),* suggesting that these SNPs influence the mechanism by which sterols regulate alternative splicing. On the basis of these findings, these SNPs would be predicted to be more strongly associated with inter-individual variation in ambient levels of plasma LDL-cholesterol than with changes induced by statin treatment. Indeed, this appears to be true since neither rs3846662 nor rs688 have been associated with statistically significant changes in LDL-cholesterol after statin treatment despite their association with baseline levels of LDL-cholesterol [3], [6], [34], [35], [36], [37], [38]. A SNP by sterol interaction is consistent with the finding of sterol-induced changes in *HMGCR* alternative splicing in the HepG2 cell line, which is heterozygous for the minor allele of rs3846662. However, the HepG2 cell line is homozygous for the rs688 “C” allele, which, based on results in lymphoblast cell lines with this genotype, would predict lack of statin suppression of %*LDLR12(−),* in direct contrast to our findings. This inconsistency may reflect cell type specific differences in the genetic regulation of *LDLR* exon 12 skipping.

*HMGCR* and *LDLR* transcriptional responses to sterols are coordinately regulated by SREBP, as indicated by the correlation of their changes in response to statin in our lymphoblast cell lines (p = 0.03, n = 24). The lack of correlation between fold changes in either total *HMGCR* or *LDLR* gene expression with *%HMGCR13(−), %LDLR12(−)* or *%LDLR4(−)* indicates that the molecular mechanisms underlying variation in the transcriptional regulation of these genes in response to sterol depletion do not also influence changes in alternative splicing. These results demonstrate that variation in transcriptional regulation is independent of variation in pre-mRNA splicing. The finding that changes in *HMGCR* exon 13 skipping occur prior to changes in gene transcription provides further evidence of independent control. In the case of prolonged exposure to extreme cholesterol depletion, such as a 24 hour incubation with statin in the absence of exogenous cholesterol, this small shift in pre-mRNA splicing to generate more *13(+)* versus *13(−)* transcript may initially help correct for small imbalances in cholesterol homeostasis prior to the stimulation of a robust transcriptional response. Furthermore, the fact that this was seen only with *HMGCR* is consistent with the extensive degree to which HMGCR activity is regulated [20], and indicates that *HMGCR* alternative splicing is subject to additional forms of regulation.

We have shown that alternative splicing contributes to coordinate regulation of genes involved in cholesterol homeostasis, including the two key regulatory genes *HMGCR* and *LDLR.* This effect is influenced by *cis-*acting SNPs that blunt the suppression of alternative splicing in response to sterol depletion. Our findings indicate that alternative splicing augments the robust transcriptional response generated by changes in cellular cholesterol status, and contributes to cellular cholesterol homeostasis under conditions of variation in sterol availability, such as statin treatment.

## Methods

### Cell Exposures

HepG2 cells were grown in MEM supplemented with 1% nonessential amino acids, 1% sodium pyruvate and 10% heat inactivated fetal bovine serum (FBS) (Hyclone). Immortalized lymphoblast cell lines were derived from the Cholesterol and Pharmacogenetics clinical trial [39], and grown in RPMI 1640 media supplemented with 10% FBS, 500 U/ml penicillin/streptomycin, and 2 nmol/L GlutaMAX [4]. IMR-90 cells were grown in high glucose DMEM supplemented 2 nmol/L GlutaMAX and 10% FBS. All culture media and supplements were obtained from Invitrogen unless otherwise indicated, and all cultures were maintained at 37°C with 5% CO_2_. Simvastatin was provided as a gift from Merck (Whitehouse Station, NJ) and was converted into 99% of the activated form (beta-hydroxy simvastatin acid) as previously described [4]. LDL-cholesterol was isolated from 250 ml of plasma from a single individual as previously described [40] using written informed consent and with approval by the Committee for the Protection of Human Subjects at Children's Hospital and Research Center Oakland. LDL-cholesterol concentration was calculated by the Friedewald equation. Cells were exposed to either conditions of sterol depletion, defined as 24 hour incubation in media supplemented with 10% lipoprotein deficient serum (LPDS) and simvastatin (concentration varied by experiment), or sterol loading, defined as 24 hour incubation in media supplemented with 10% LPDS and either 50 µg/ml LDL-cholesterol or 1 µg/ml 25-hydroxycholesterol.

### Animal studies

Wild caught feral adult male St. Kitts vervets (*Cercopithecus aethiops sabaeus*), also called African green monkeys [41] were studied in two separate experiments. Because the plasma cholesterol response to dietary cholesterol varies widely among individuals, plasma cholesterol response to a 4 week challenge with a diet containing 35% of energy as saturated fat and 0.4 mg/kcal of dietary cholesterol was assessed for each animal. A 4 month washout with monkey chow diet followed this initial diet challenge. In the first experiment, animals were fed a diet with no added cholesterol (see Rudel *et al.* [42] for diet composition) for 22 weeks, n = 20. Animals were then fed for 19-weeks with the same diet supplemented with 0.6 mg/kcal cholesterol with either 35% of the energy as monounsaturated fat (n = 10) or saturated fat (n = 10). Liver biopsies were surgically collected via a midline laparotomy before and after cholesterol feeding, and tissues were stored at −80°C until analysis. A portion of frozen tissue was taken, weighed, and liver lipid concentrations were determined after lipid extraction using the enzymatic methods previously described [43]. One animal died before liver biopsies were collected and data from a second animal was omitted since it demonstrated abnormal transcriptional up-regulation of *HMGCR* and *LDLR* after cholesterol feeding. In the second experiment animals were fed a diet containing 35% of energy as monounsaturated fat supplemented with 0.002, 0.2 or 0.4 mg/kcal cholesterol (n = 5 per diet), and liver biopsies were collected after 10-weeks. All procedures were approved by the Wake Forest University Animal Care and Use Committee through protocols A04-048 and A10-024. All efforts were made to minimize suffering using the appropriate anesthetic and analgesic agents. Animals were routinely monitored by veterinarians for all medical and dental conditions, with appropriate treatments administered by specialists.

Liver tissue was snap frozen in liquid nitrogen at the time of collection and was stored at −80°C until processed for RNA extraction. Liver RNA was extracted with Trizol (Invitrogen), and RNA concentration and integrity was verified by A260/280 reading and gel electrophoresis. Expression of alternatively spliced transcripts was verified by RT-PCR and DNA sequencing; primer sequences are listed in **Table S1**. Positively identified splice variants were quantified by qPCR. Baseline quantities of each transcript were calculated as the average of experiment 1 animals collected before cholesterol feeding (n = 18) and experiment 2 animals after 0.002 mg/kcal cholesterol feeding (n = 5). Transcript fold changes with cholesterol feeding for each individual were calculated as the transcript quantity after cholesterol feeding divided by the averaged baseline value (n = 28). Since there were no consistent statistically significant differences in cholesterol-induced changes in *%HMGCS1 2(−), %MVK4(−), %LDLR4(−), %LDLR12(−)* or %*PCSK9 8(−)* among the three cholesterol doses, the data for all doses were combined for each of these splice variants.

### siRNA transfection

*PTBP1* knock-down was achieved by 18 hour transfection of 2.5×10^5^ HepG2 cells/well in 6-well plates with 12.5 pmole Silencer Select siRNA (Applied Biosystems) using pSPORTNeoFX transfection agent (Applied Biosystems) following the manufacturer's protocol. Cells were exposed in replicate to either the *PTBP1* siRNA duplex, the Silencer Select Negative Control #1 (Applied Biosystems), pSPORTNeoFx transfection agent only, or no additions to ensure that changes in alternative splicing with knock-down were specific to the reduction of *PTBP1*. Knock-down was confirmed by qPCR and Western blot incubated with a monoclonal mouse anti-PTB antibody (Invitrogen) and GAPDH (D-6) mouse monoclonal antibody (Invitrogen). Protein band intensity was quantified on the Alpha Imager TM.

### Transcript quantification

RNA was extracted using the Qiagen RNAeasy (Qiagen) mini-kit with QIAshredders, and 5 µg of RNA was reverse transcribed into cDNA using the Applied Biosystems cDNA archive kit. Specific qPCR assays were designed using Primer3 [44]. For all assays, either one primer (Elim Biopharmaceuticals) or the fluorescent probe (Applied Biosystems) was designed to directly overlay the site of alternative splicing, for example the exon 3 to exon 5 splice junction in the case of *LDLR4(−)*. All transcripts were quantified in both human and monkey samples with either TaqMan or SYBR Green assays using TaqMan or SYBR Universal Master Mix (Applied Biosystems) with primer sequences listed in **Table S1**. *PCSK9* and *HMGCR* splice variants were quantified using the assays previously described [4], [5]. The remaining splice variants were quantified using SYBR Green Master Mix, with dissociation curves run at the end of each reaction to ensure the generation of a single PCR amplicon. All reactions were performed in triplicate using 50 ng cDNA per reaction. Absolute quantification of splice variants was performed using a serially diluted standard containing the same sequence as the target amplicon. The specificity of all qPCR assays was verified by testing for detection of a standard known to contain only the full-length transcript of a gene with the qPCR assay specific for its corresponding alternatively spliced variant (for example, *HMGCR13(+)* template with *13(−)* qPCR assay). Lack of cross-reaction in the reverse direction (for example, *HMGCR13(−)* template with *13(+)* qPCR assay) was also confirmed. Primer and probe sequences are listed in **Table S2**. CLPTM1 and SLC7A were quantified in all samples for data normalization. For Figure 1A only, both *HMGCR13(+)* and *13(−)* transcripts were amplified by PCR using the following primers, HMGCRex12.F: tgctaagcatatcccagcctacaag and HMGCRex14.R: atgcctcctttatcactgcgaacc. PCR product was loaded onto an agarose gel, and band density was quantified using the AlphaView Software 1.2.01 (Alpha Innotech).

Percent alternatively spliced mRNA was calculated as the quantity of the splice variant divided by the quantity of the total transcripts per gene, e.g., 100× *HMGCR13(−)* divided by *[HMGCR13(−) + HMGCR13(+)].* Fold changes were calculated as the value of the percent alternatively spliced after statin treatment or sterol loading, divided by the percent alternative spliced under basal culture conditions, i.e., %*HMGCR13(−)* with statin + 10% LPDS divided by %*HMGCR13(−)* +10% FBS. All experiments were performed in triplicate unless otherwise indicated.

### Measurement of transcript half-life

HepG2 (n = 12) were first incubated for 24 hours with either 2.0 µM simvastatin + 10% LPDS or sham buffer, after which 1 µg/ml actinomycin D was added. Samples were removed at eight time points over the course of 48 hours, and splice variants were quantified as described above. Transcript half-life was calculated as previously described using only time points consistent with first order decay kinetics [45]. Half-life was calculated from each individual experiment with t-tests used to compare the mean and standard error values between statin versus sham incubated cells. In addition, mRNA data per time point was averaged, and half-life was calculated from the pooled data. Half-life values measured in the averaged versus pooled data sets were not significantly different. To determine the effects of PTBP1 on transcript half-life, HepG2 cells were transfected with either a non-targeting siRNA control or PTBP1 specific siRNA as previously described, n = 12. After 18 hours, 1 µg/mL actinomycin D was added, and transcript half-life was measured as described above.

### Genotyping

Rs3846662 genotyping was performed as previously described [34]. Rs688 genotyping was performed using a fluorogenic allele-specific amplification method (Millipore) as previously described [6]. Following amplification, fluorescence was read using an ABI 7900HT (Applied Biosystems) and cluster analysis performed using SDS v2.3 software (Applied Biosystems).

### Statistical Analyses

qPCR data were analyzed as previously described with all data normalized to CLPTM1 and SLC7A, whose expression was validated to be non-responsive to sterols [4]. For gene expression quantification by qPCR, the Grubb's test for outliers was calculated for each three triplicate measurement. For dose response curves, repeated measures MANOVA was used to assess statin-induced differences between the up-regulation of the *HMGCR13(+)* and *13(−)* transcripts. Statistically significant differences in percent alternatively spliced transcripts (*HMGCR, LDLR, HMGCS1, MVK,* or *PCSK9)* after *in vitro* treatment with simvastatin, LDL-cholesterol or 25-hydroxycholesterol were calculated using two-tailed paired t-tests. Two-tailed paired t-tests were also used to identify statistically significant differences in the magnitude of change between the full length and alternatively spliced transcripts in cholesterol versus control fed monkeys. Correlations between %*HMGCR13(−)* with *%LDLR12(−)* and *%LDLR4(−)* among statin or 25-hydroxycholesterol treated immortalized lymphoblast cell lines were assessed by linear regression. To test for a significant interaction between rs3846662 with statin suppression of *%HMGCR13(−),* multivariate regression models were created with *%HMGCR13(−)* after statin as the dependent variable, and *%HMGCR13(−)* at baseline as the independent variable with adjustment for rs3846662 and an interaction between *%HMGCR13(−)* at baseline with rs3846662. A similar model was generated to assess interaction between rs688 and statin suppression of *%LDLR12(−).* All statistical analyses were performed using JMP 7.0.1 (SAS Institute).

## Supporting Information

Figure S1 **Change in hepatic total cholesterol and cholesterol ester is correlated with change in percent LDLR alternative splicing.** Hepatic total cholesterol and cholesterol ester were measured in liver biopsies obtained from African green monkeys with (n = 19) and without (n = 14) cholesterol supplementation. Percent change in hepatic lipids for each cholesterol fed animal were calculated from the average of all control fed animals, and values were adjusted for the change in total plasma cholesterol as well as the predominant fat (monounsaturated versus saturated) in each diet to account for differences in response due to variation in the amount of cholesterol supplementation (0.2, 0.4 and 0.6 kcal/g). Direct correlation in inter-individual variation in the residuals of percent change in hepatic lipids with change in alternative splicing was assessed in JMP 7.0.1. Animals who experienced greater increases in hepatic total cholesterol (**A**) and cholesterol ester (**B**) also had greater increases in *%LDLR12(-).* Similar relationships were seen with other splice variants, but did not achieve statistical significance. There was no relationship between change in hepatic free cholesterol and *%LDLR12(-).*This lack of relationship is consistent with the fact that levels of hepatic free cholesterol were not elevated in a statistically significant manner after cholesterol feeding (3.0±0.1 mg/g liver no cholesterol versus 4.2±0.5 mg/g liver with cholesterol, p = 0.07), compared to changes in hepatic cholesterol ester (2.4±0.5 mg/g liver no cholesterol versus 15.6±2.7 mg/g liver with cholesterol, p<0.001). **Scale reflects residual percent change values after adjustment as described above.
(TIF)

Figure S2 **Effect of PTBP1 knock-down on transcript half-life.** Actinomycin D (1µg/ml) was added to HepG2 cells after 18hr transfection with either PTBP1 Silence Select siRNA or a non-targeting siRNA control, n = 8. All values shown are mean ± s.e.m. *p<0.05, half-life is significantly different between cells transfected with the non-targeting siRNA and the PTBP1 specific siRNA.
(TIF)

Figure S3 **Change in total transcript levels with PTBP1 knock-down.** HepG2 cells were transfected with either a siRNA targeted to PTBP1 or a non-targeting negative control in duplicate. After 18 hours, incubation media was refreshed to include either 2.0µM simvastatin + 10% LPDS or placebo buffer + 10% FBS and cells were incubated for an additional 24 hours, n = 8. Statin induced fold changes in gene expression were calculated independently in the PTBP1 siRNA versus non-targeting negative control samples as the value measured in the statin incubated sample divided by the value measured in the placebo incubated sample. All values shown are mean ± s.e.m.
(TIF)

Table S1 **Primer and probe sequences used for quantitative real time PCR to detect specific splice variants.** All assays with probe sequences listed were used as TaqMan assays, assays without a probe sequence were used as SYBR Green assays.
(DOC)

Table S2 **Primer sequences used to detect expression of alternatively spliced transcripts in the African Green Monkey.**
(DOC)
