# Supplementary figures and images for: Coordinately Regulated Alternative Splicing of Genes Involved in Cholesterol Biosynthesis and Uptake
Source: PLoS One. 2011 Apr 29;6(4):e19420. doi: 10.1371/journal.pone.0019420 (PMC3084847; doi:10.1371/journal.pone.0019420)

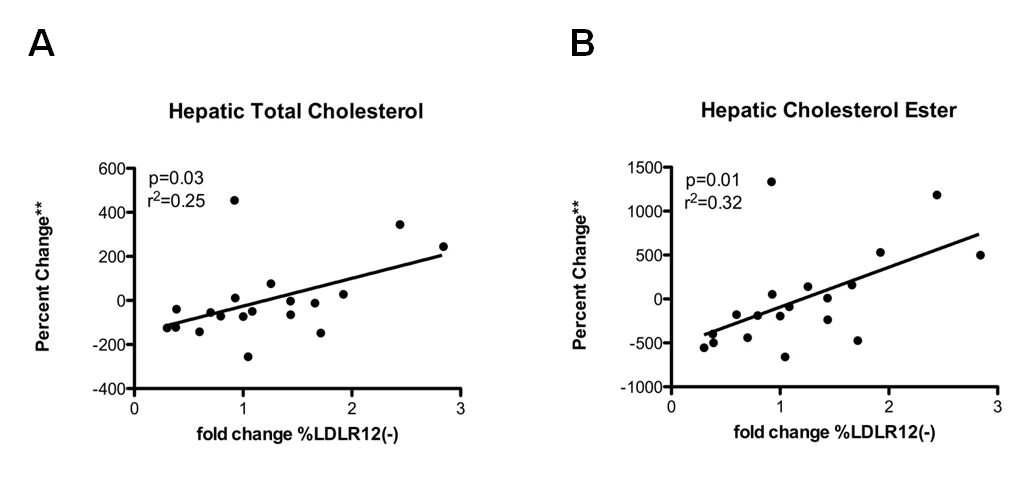

Supplement: Figure S1 — Change in hepatic total cholesterol and cholesterol ester is correlated with change in percent LDLR alternative splicing. Hepatic total cholesterol and cholesterol ester were measured in liver biopsies obtained from African green monkeys with (n = 19) and without (n = 14) cholesterol supplementation. Percent change in hepatic lipids for each cholesterol fed animal were calculated from the average of all control fed animals, and values were adjusted for the change in total plasma cholesterol as well as the predominant fat (monounsaturated versus saturated) in each diet to account for differences in response due to variation in the amount of cholesterol supplementation (0.2, 0.4 and 0.6 kcal/g). Direct correlation in inter-individual variation in the residuals of percent change in hepatic lipids with change in alternative splicing was assessed in JMP 7.0.1. Animals who experienced greater increases in hepatic total cholesterol (A) and cholesterol ester (B) also had greater increases in %LDLR12(-). Similar relationships were seen with other splice variants, but did not achieve statistical significance. There was no relationship between change in hepatic free cholesterol and %LDLR12(-).This lack of relationship is consistent with the fact that levels of hepatic free cholesterol were not elevated in a statistically significant manner after cholesterol feeding (3.0±0.1 mg/g liver no cholesterol versus 4.2±0.5 mg/g liver with cholesterol, p = 0.07), compared to changes in hepatic cholesterol ester (2.4±0.5 mg/g liver no cholesterol versus 15.6±2.7 mg/g liver with cholesterol, p<0.001). **Scale reflects residual percent change values after adjustment as described above. (TIF) [file pone.0019420.s001.tif]

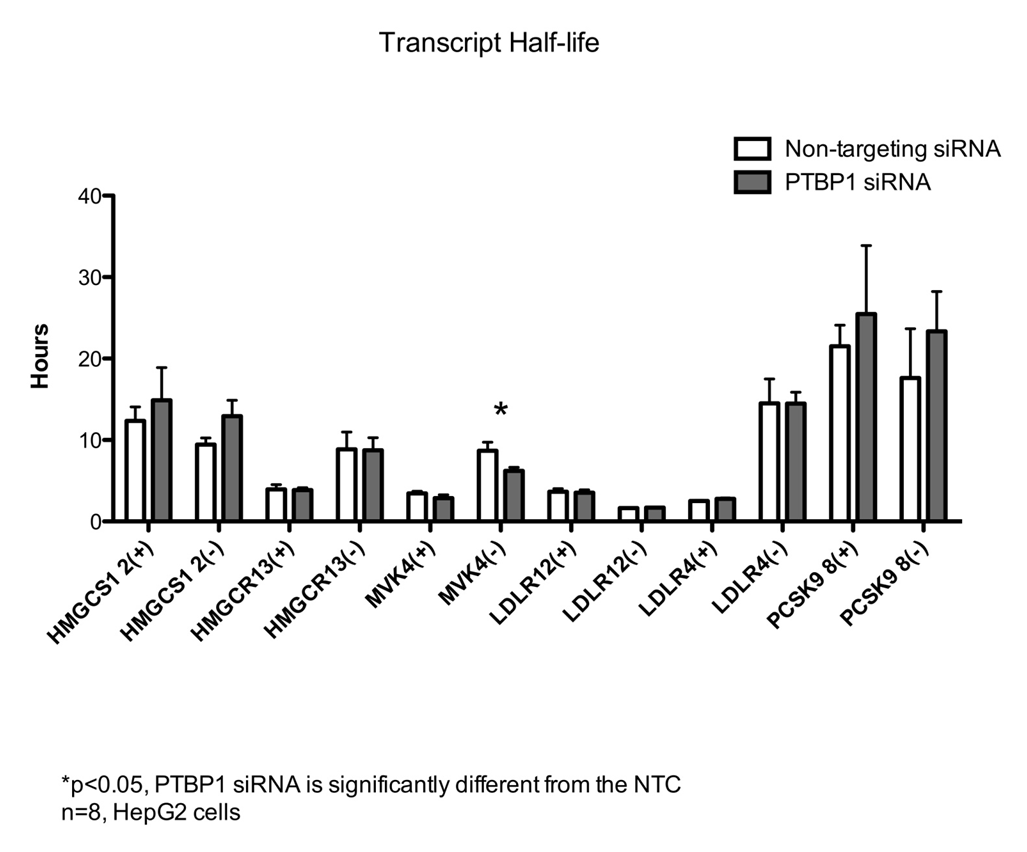

Supplement: Figure S2 — Effect of PTBP1 knock-down on transcript half-life. Actinomycin D (1µg/ml) was added to HepG2 cells after 18hr transfection with either PTBP1 Silence Select siRNA or a non-targeting siRNA control, n = 8. All values shown are mean ± s.e.m. *p<0.05, half-life is significantly different between cells transfected with the non-targeting siRNA and the PTBP1 specific siRNA. (TIF) [file pone.0019420.s002.tif]

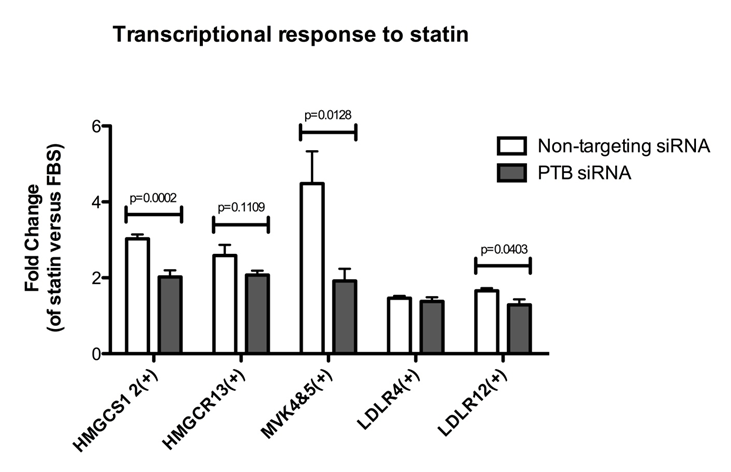

Supplement: Figure S3 — Change in total transcript levels with PTBP1 knock-down. HepG2 cells were transfected with either a siRNA targeted to PTBP1 or a non-targeting negative control in duplicate. After 18 hours, incubation media was refreshed to include either 2.0µM simvastatin + 10% LPDS or placebo buffer + 10% FBS and cells were incubated for an additional 24 hours, n = 8. Statin induced fold changes in gene expression were calculated independently in the PTBP1 siRNA versus non-targeting negative control samples as the value measured in the statin incubated sample divided by the value measured in the placebo incubated sample. All values shown are mean ± s.e.m. (TIF) [file pone.0019420.s003.tif]
