# Supplementary material for: Coordinately Regulated Alternative Splicing of Genes Involved in Cholesterol Biosynthesis and Uptake
Source: PLoS One. 2011 Apr 29;6(4):e19420. doi: 10.1371/journal.pone.0019420 (PMC3084847; doi:10.1371/journal.pone.0019420)
Supplement: Table S1 — Primer and probe sequences used for quantitative real time PCR to detect specific splice variants. All assays with probe sequences listed were used as TaqMan assays, assays without a probe sequence were used as SYBR Green assays. (DOC) [file pone.0019420.s004.doc]

Supplementary Table 1. Primers used for qPCR.

| **Transcript** | **Forward Primer** | **Reverse Primer** | **Probe** |
| --- | --- | --- | --- |
| HMGCS1 2(+) | ccgaaggaggaaacagtgac | ggcaacaattcccacatctt |  |
| HMGCS1 2(-) | tcacgcttgctctttcaccatgcc | ctatactttccagcatctacaccatca |  |
| MVK 4(+) | tgatgtcacaacacccacct | cggcagatggacaggtataa |  |
| MVK 4(-) | ctttctggggccctgccg | gatctcctcgcacacagtca |  |
| LDLR 4(+) | aaggctgtccccccaaga | gagctgttgcactggaagc | ctgccacgatggga |
| LDLR 4(-) | cattaacgcagccaacttcatc | aaggctgtcctgtggccac | agtgctctgatggaaac |
| LDLR 12 (+) | atcaccctagatctcctcagtg | gcactgaaaatggcttcgtt |  |
| LDLR 12 (-) | ggcatcaccctaggacaaagt | gggtgaggttgtggaagagaa |  |
| PCSK9 8(+) | acgtggctggcattgca | aagtggatcagtctctgcctcaa |  |
| PCSK9 8(-) | gtgggctggttgcagctgtt | tcccactcctggagaaactggag |  |
| PTBP1 | aggtcaccaacctcctgatg | tgtggttggagaactggatg |  |
