# Supplementary material for: Coordinately Regulated Alternative Splicing of Genes Involved in Cholesterol Biosynthesis and Uptake
Source: PLoS One. 2011 Apr 29;6(4):e19420. doi: 10.1371/journal.pone.0019420 (PMC3084847; doi:10.1371/journal.pone.0019420)
Supplement: Table S2 — Primer sequences used to detect expression of alternatively spliced transcripts in the African Green Monkey. (DOC) [file pone.0019420.s005.doc]

**Supplementary Table 2. Primers used for RT-PCR.**

| **Gene** | **Exons Spanned** | **Forward Primer** | **Reverse Primer** |
| --- | --- | --- | --- |
| HMGCS | 1 to 3 | tggcggctataaagctggt | ggcaacaattcccacatgtt |
| HMGCR | 12 to 14 | cccagcctacaagttggaaa | gagccaggctttcacttctg |
| MVK | 3 to 6 | ggacctcagcttacccaaca | agctccaaatcctccttggt |
| LDLR | 3 to 5 | gctgcattcctcagttctgg | cagagcactggaattcgtca |
| LDLR | 11 to 14 | aactcccgccaagatcaag | cctctcacaccagttcactcc |
| PCSK9 | 7 to 9 | actgcagcacctgctttgt | gtgtaggccccgagtgtg |
